# Treatment of pre-collapse non-traumatic osteonecrosis of the femoral head through Orthopdische Chirurgie München approach combined with autologous bone mixed with β-tricalcium phosphate porous bioceramic bone graft: a retrospective study of mid-term results

**DOI:** 10.1186/s13018-021-02632-x

**Published:** 2021-08-12

**Authors:** Dawei Liang, Jia Pei, Leilei Zhang, Haonan Ling, Youwen Liu, Xiantao Chen

**Affiliations:** 1 Hip Disease Research Center, Luoyang Orthopedic-Traumatological Hospital of Henan Province (Henan Provincial Orthopedic Hospital), 82 Qiming South Road, Luoyang, 471000 Henan China; 2 Quality Management Department, Luoyang Orthopedic-Traumatological Hospital of Henan Province (Henan Provincial Orthopedic Hospital), Luoyang, 471000 China

**Keywords:** Osteonecrosis of the femoral head, Bone grafting, OCM approach, Non-vascularized, Risk factors, THA

## Abstract

**Background:**

This study aimed to evaluate the clinical efficacy of femoral head and neck fenestration combined with autologous bone mixed with β-tricalcium phosphate porous bioceramic bone (light bulb procedure) through Orthopdische Chirurgie München approach (OCM approach) for pre-collapse non-traumatic osteonecrosis of the femoral head(ONFH).

**Methods:**

The clinical data of 47 patients (47 hips) with ONFH were retrospectively reviewed. The Harris hip score (HHS) was used to evaluate the clinical outcomes. Imaging was assessed by X-ray. Clinical failure was defined as postoperative total hip arthroplasty (THA) or the HHS was poor (< 70). The Kaplan–Meier survival curve was used to conduct a univariate analysis of risk factors. The analysis factors included gender, age, International Association Research Circulation Osseous (ARCO) stage, etiology, body mass index (BMI), 25-hydroxyvitamin D (25(OH)D), and type I collagen carboxy-terminal peptide (CTX). The COX multivariate risk model was used to analyze the risk factors.

**Results:**

All the 47 hips were followed up for 24–58 months, with an average of 45 months. The Harris score (76.29 ± 10.38) at the last follow-up was significantly higher than the preoperative HHS (64.45 ± 2.93) (*P* < 0.05). The postoperative HHS was excellent with a success rate of 36.17%. Postoperative imaging evaluation showed that 9 hips improved, 28 hips stabilized, and 10 hips progressed. Moreover, 17 out of 47 hips were defined as a postoperative clinical failure and the success rate was 63.83%. 25(OH)D and preoperative ARCO stage were risk factors for postoperative clinical failure (*P* < 0.05). The COX multivariate risk model analysis showed that IIIA stage was an independent risk factor for postoperative clinical failure (*P* < 0.05).

**Conclusions:**

The head and neck fenestration and bone grafting via the OCM approach in the treatment of non-traumatic ONFH in the pre-collapse stage can achieve good clinical outcomes. 25(OH)D deficient patients and ARCO IIIA patients had a higher failure rate of bone graft using this approach.

## Introduction

Osteonecrosis of the femoral head (ONFH) is a frequent and refractory disease in the field of orthopedics that still requires comprehensive exploration. ONFH is a decrease in blood flow to the femoral head, which causes partial death of bone cells and bone marrow components, followed by repair. It subsequently causes osteonecrosis, resulting in structural changes in the femoral head and even collapse, causing pain and dysfunction of the hip joint [1, 2]. ONFH is most common in 20- to 50-year-old patients. The number of new patients in the USA is about 20,000–30,000 per year. Besides, 2 of 100,000 patients in the UK suffer from ONFH [3, 4]. New cases are increasing at 150,000–200,000 per year in China. Common causes of ONFH include alcohol, glucocorticoids, trauma, pregnancy, blood diseases, and organ transplant [5, 6]. ONFH patients usually suffer from femoral head collapse without intervention. Therefore, it is necessary to undergo short-term total hip arthroplasty (THA). However, young patients undergoing primary THA may face several revision challenges [7]. If effective treatment can be received in the early stage of ONFH, THA may be delayed or even avoided.

Currently, the methods used to preserve joint treatment include conservative and surgical treatments. Conservative treatment includes pulsed electromagnetic fields, extracorporeal shock wave and drug therapy [8]. However, conservative treatment can relieve pain but cannot prevent the process of necrosis, hence the effect is limited. Surgical treatments include core decompression, platelet-rich plasma or bone marrow mesenchymal stem cells, vascularized bone graft, non-vascularized bone graft, and osteotomy [9–11]. Core decompression is accepted by most orthopedic surgeons as the preferred option for the treatment of ONFH but with wide variation in clinical outcomes [12]. Non-vascularized bone grafting is also an effective treatment for early-stage ONFH [13]. For example, through the femoral head window to remove necrotic bone tissue, autologous bone mixed with β-tricalcium phosphate porous bioceramic bone grafting, promotion of bone formation, strengthening of the internal structure of the femoral head, and avoidance of collapse. The Orthopdische Chirurgie München approach (OCM approach) enters through the space between the tensor fascia lata and gluteus medius muscle to reduce soft tissue damage, preserve the joint capsule and tendon stops, and reveal the head and the neck. The proximal end through this approach allows direct access to the ilium as a bone grafting material, reducing the surgical incision. It is also used in THA as a minimally invasive approach. However, the use of this approach for non-vascularized bone grafting has not been reported in recent years. The present study aimed to evaluate the results of the OCM approach through the functional recovery quality, clinical results, imaging, and survival rate of patients with non-traumatic ONFH before the collapse.

## Materials and methods

### Clinical data

This study was approved by the medical ethics committee of the Luoyang Orthopedic-Traumatological Hospital of Henan Province (Henan Provincial Orthopedic Hospital) institution (20,160,301). Informed consent was obtained from all participants. From January 2016 to December 2016, 47 patients (47 hips) with non-traumatic ONFH in pre-collapse stage were treated with autologous bone mixed with β-tricalcium phosphate porous bioceramic bone grafting through OCM approach. There are 39 males and 8 females, aged 24–50 years old, with an average of 38 years old. According to the etiology, it is classified as alcoholic, hormonal, and idiopathic. Preoperative magnetic resonance imaging (MRI), pelvic position, and frog radiograph were performed. The diagnostic criteria of ONFH were according to the International Association Research Circulation Osseous (ARCO) criteria [14]. General information is shown in Table 1.

**Table 1:** Demographic characteristics of patients

Parameter	Numerical value	Statistics
Gender		
male	39	
female	8	
Age(y)	38.3 ± 7.5	
BMI (kg/m^2^)	25.9 ± 2.9	
25(OH)D (nmol/L)	39.6 ± 17.9	
CTX (ng/mL)	0.828 ± 0.418	
Etiology		
Alcoholic	21	
Hormonal	13	
Idiopathic	13	
ARCO stage		
ARCO IIB	10	
ARCO IIC	14	
ARCO IIIA	23	
Follow up time (month)	44.6 ± 10.0	
Operative time (min)	116.70 ± 14.19	
Intraoperative blood loss (ml)	220.64 ± 75.63	
Harris hip score		
Preoperative	64.45 ± 2.93	*P* < 0.001
Postoperative	76.29 ± 10.38	

### Surgical methods

All surgical operations were performed by the same orthopedic team.

The bone graft was selected from autologous iliac bone mixed with β-tricalcium phosphate porous bioceramic bone; the ratio is 1:1. Autologous bone transplantation was taken from the patient’s iliac bone: a 2–4 cm incision was made along the iliac crest at about 5 cm behind the anterior superior iliac spine, the periosteum and attached muscles were separated, and the osteotome tomes took about 1–3 cm long of the iliac semi-cortical bone plate and sutured the donor area.

β-Tricalcium phosphate porous bioceramic bone is provided by Shanghai bio-lu Biomaterial Co., Ltd. The shape is granular, 1.0–3.5 mm, the pore diameter is 100–500 µm, the porosity is (70 ± 15)%, the pore communication rate is 100%, and the mechanical strength reaches 45 Mpa.

After anesthesia, patients were placed in the supine position with the operated sacral coccyx raised on the surgical side. The incision in the middle section connecting the anterior superior iliac spine of the hip and the anterior edge of the greater trochanter was about 8 cm long. The skin, subcutaneous fascia, and the broad fascia along the anterior edge of the gluteus medius muscle, blunt separation between gluteus medius and tensor fasciae latae. The anterolateral joint capsule of the hip joint was exposed and the joint capsule was cut longitudinally. Narrow acetabular retractors were placed on the upper and the lower sides of the femoral neck to expose the junction of the femoral head and the neck. A 10 × 8-mm bone window was made at the head-neck junction and an angled osteotome was used to clean the dead bone in the femoral head, curettage was performed with a curette, and the subchondral bone was preserved. Autologous bone mixed with β-tricalcium phosphate porous bioceramic bone grafting were implanted into the defect in the femoral head, the bony block at the original opening window closed the bone window, the articular capsule was repaired, and the tissue was stitched repeatedly after washing the wound.

The postoperative functional exercise was dominated by muscle isometric contraction. After 6 weeks of operation, a double crutch was avoided to prevent limb loading. In the subsequent 6 weeks, the lower limbs protected by crutches gradually increased the weight. Patients subsequently slowly discarded the crutches to progress to full weight-bearing within 3–6 months after operation, according to the radiographic evaluation of the femoral head. But avoided jumping and strenuous activity.

The Harris hip score (HHS) was used to assess pain, joint deformity, range of motion, and joint function. A score less than 70 was classified as poor, 70–80 as fair, 80–90 as good, and 90–100 as excellent. Patients were regular follow-up at 3, 6, and 12 months, and annually thereafter. Patients undergoing THA during follow-up, preoperative HHS was then included in the evaluation, and follow-up assessment no longer participate. Anteroposterior pelvic and frog radiographs were taken for analysis. The imaging analysis was completed by the same senior doctor. For imaging evaluation, stable femoral head morphology and partial or complete repair of the necrotic area were considered to be improving. Stable was defined as the unchanged necrotic area of the femoral head. Progressive collapse of the femoral head, expansion of necrotic areas, joint space narrowing, or arthritis were considered to be progressing. Clinical failure was defined as postoperative THA or poor HHS.

### Statistical analysis

SPSS 19.0 software (Chicago, IL) was used for analysis. The chi-square test was used for comparison of count data between groups and measurement data were expressed as mean ± standard deviation. A paired *t* test was used for comparison before and after surgery and *P* < 0.05 was considered statistically significant. The Kaplan–Meier survival curve was used for univariate analysis of risk factors. Risk factors included gender, age, ARCO stage, etiology, body mass index (BMI), 25-hydroxyvitamin D (25(OH)D), and type I collagen carboxy-terminal peptide (CTX). The Cox multivariate risk model was used for multivariate analysis of risk factors.

## Results

**Fig. 1:**
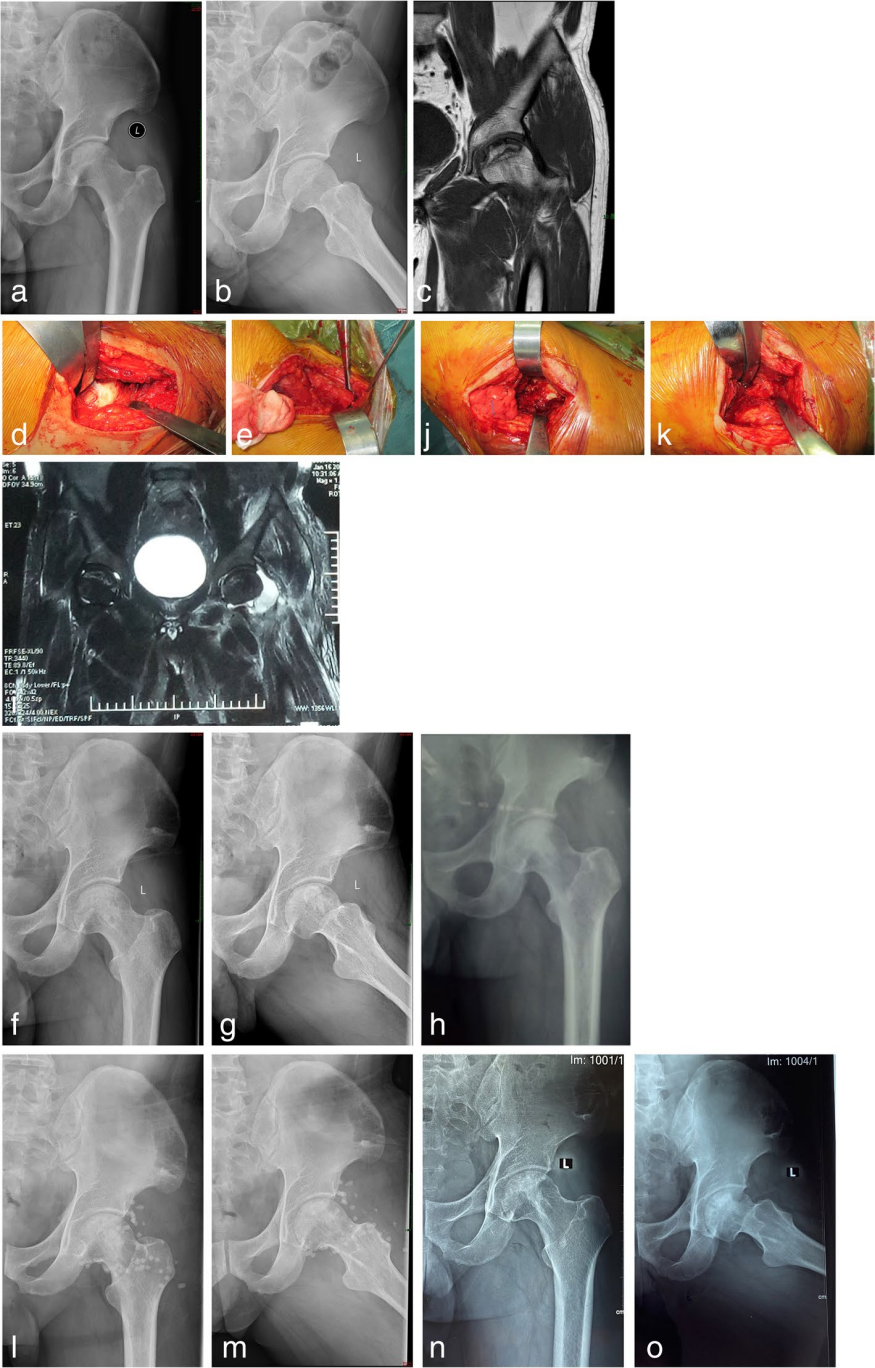
**a**, **b** Anteroposterior and frog-like radiographs of a 38-year-old male patient showing uneven density within the femoral head. **c** MRI T1WI showed femoral head necrosis. **d** OCM approach fenestration in the head and neck images of non-vascularized bone graft blocks occluding the bone window (black arrow). **e** Images of the ilium taken with subcutaneous tissue detached proximally from the same incision (black arrow). **f**, **g** Anteroposterior and frog radiographs of the hip at 3 months postoperatively showed increased bone density in the femoral head and good osteogenesis in the bone graft area. **h** At 6 months post-operation, the frontal radiograph of the hip joint showed good osteogenesis in the femoral head and swelling of the soft tissue around the hip due to high fever and pain in the operation area. **i** MRI T2WI showed synovial hyperplasia and edema in the joint. Edema of the muscle layer around the hip joint may indicate infection. **j** Original incision exposed the neck of the femoral head to visible intra-articular purulent secretions (black arrow). **k** Bone healing at the fenestration in the neck of the original femoral head (black arrow). **l**, **m** Anteroposterior and frog radiographs of the hip after debridement. **n**, **o** Anteroposterior and frog radiographs of the hip at the last follow-up show good osteogenesis of the bone graft region and stable femoral head

All 47 patients were followed up. The follow-up time was 24–58 months, with an average of 45 months. The average intraoperative blood loss was 220.64 ± 75.63 ml and the average operation time (the time from skin incision to the end of suture) was 116.70 ± 14.19 min. Lateral femoral cutaneous nerve injury occurred in one case. One case of hypoproteinemia developed an infection at 6 months after surgery due to the long-term nutritional inadequacy. The bony healing at the originals fenestration of the femoral head neck was seen during debridement surgery. At the end of follow-up, the X-ray showed good ossification in the femoral head and the HHS was excellent (Fig. 1). The HHS of the 47 patients at the last follow-up was (76.29 ± 10.38), which was significantly different from the preoperative score (64.45 ± 2.93, *t* = 8.116, *P* = 0.000). The postoperative HHS was excellent in 7 hips, good in 10 hips, fair in 16 hips, and poor in 14 hips, with an excellent and good rate of 36.17%. Imaging evaluation showed an improvement of 9 hips, stabilization of 28 hips, and progress of 10 hips. Postoperatively defined as clinical failure of 17 hips, of which 12 hips have undergone THA. Although the remaining 5 hips had poor HHS, the patient did not undergo THA according to the patient’s wishes and is still under follow-up observation. The Kaplan–Meier survival curve of single-factor analysis of risk factors showed that 25(OH)D and ARCO stage were risk factors for postoperative clinical failure (*P* < 0.05) (Table 2). The COX multivariate risk model analysis showed that the ARCO IIIA stage was an independent risk factor for postoperative clinical failure (*P* < 0.05) (Table 3). Table 4 shows the demographic characteristics of patients with ARCO IIIA.

**Table 2:** Single factor analysis of risk factors for postoperative failure

Variable	Clinical success	Clinical failure	Statistic
Gender			
Male	25	14	χ^**2**^ = 0.011
Female	5	3	*P* = 0.915
Age(y)			
< 40	19	7	χ^**2**^ = 1.748
≥ 40	11	10	*P* = 0.186
BMI (kg/m^2^)			
< 24	5	6	χ^**2**^ = 1.964
≥ 24	25	11	*P* = 0.161
25(OH)D (nmol/L)			
< 50	17	15	χ^**2**^ = 3.934
≥ 50	13	2	*P* = 0.047
CTX (ng/mL)			
< 0.714	12	4	χ^**2**^ = 0.900
≥ 0.714	18	13	*P* = 0.343
Etiology			
Alcoholic	16	5	χ^**2**^ = 2.049 *P* = 0.359
Hormonal	7	6	
Idiopathic	7	6	
ARCO stage			
ARCO IIB	9	1	χ^**2**^ = 11.293 *P* = 0.004
ARCO IIC	12	2	
ARCO IIIA	9	14

**Table 3:** COX multivariate risk model analysis of risk factors for postoperative clinical failure

Variable	Wald χ^**2**^	Relative risk	95%CI		*P* value
			lower	upper	
ARCO stage					
ARCO IIB					
ARCO IIC	2.598	0.175	0.021	1.458	0.107
ARCO IIIA	4.347	0.204	0.046	0.909	0.037
25(OH)D (nmol/L)					
< 50	1.009	2.209	0.471	10.364	0.315
≥ 50

**Table 4:** Demographic characteristics of patients with ARCO IIIA

	Gender	Age(y)	Etiology	BMI (kg/m^2^)	25(OH)D (nmol/L)	CTX (ng/mL)	Follow up time (month)
Clinical failure	Male	50	Alcoholic	23	12.19	0.958	31
	Male	50	Idiopathic	26	17.85	0.147	28
	Male	50	Alcoholic	21	20.76	1.232	28
	Male	41	Idiopathic	23	21.17	0.739	48
	Male	49	Hormonal	23	23.78	1.005	30
	Male	32	Idiopathic	29	26.77	1.319	44
	Male	47	Alcoholic	24	32.05	0.730	40
	Male	43	Idiopathic	29	32.40	0.890	37
	Female	39	Idiopathic	22	40.14	0.922	30
	Male	29	Hormonal	32	74.01	0.144	24
	Female	47	Hormonal	28	45.40	1.203	50
	Male	35	Hormonal	25	47.71	0.942	24
	Male	34	Hormonal	25	24.37	0.890	36
	Male	46	Idiopathic	29	20.76	1.353	40
Clinical success	Male	40	Alcoholic	27	15.99	0.531	56
	Male	40	Alcoholic	28	18.23	1.241	58
	Male	31	Alcoholic	28	54.23	0.145	48
	Male	37	Idiopathic	22	54.59	0.148	52
	Male	32	Alcoholic	34	34.41	1.364	52
	Male	32	Alcoholic	29	38.43	1.430	53
	Male	30	Idiopathic	25	40.14	1.181	41
	Female	24	Hormonal	22	45.40	0.837	54
	Female	49	Hormonal	28	72.64	1.069	39

## Discussion

ONFH is a common disease in young or active patients and may be related to alcoholism, hormones, trauma, or other risk factors. For young patients, long-term efficacy and years of use of THA are still fraught with uncertainty; therefore, preserving the patient’s joints is of great importance. Current interventions include core decompression, various types of osteotomies, and vascularized or non-vascularized bone grafts [2]. Core decompression is a popular treatment for early ONFH but it cannot effectively remove all dead bones and cannot provide support for subchondral bone [9]. Osteotomy changes the anatomy of the proximal femur, which may cause difficulties for THA in the future. In addition, it has a higher complication rate, including the non-union or delayed union of bone at the osteotomy, failure of internal fixation, etc. [11, 15, 16]. Vascularized bone grafts require a high surgical technique, long operation time, extensive trauma, and have highly variable long-term success rates [17, 18]. Therefore, many orthopedic surgeons choose non-vascularized bone grafts to treat ONFH, such as fenestration of the femoral head and neck (bulb technique).

In the head and neck fenestration technique, the OCM approach was utilized to treat pre-collapse femoral head necrosis. The average intraoperative blood loss was 220.64 ± 75.63 ml and the average operation time was 116.70 ± 14.19 min, which had certain advantages. The OCM approach is a modification of the Watson-Jones approach. As a minimally invasive approach, it enters through the gap between the gluteus medius muscle and the tensor fascia lata muscle to reduce muscle damage [19]. This approach has the advantages of less trauma, less blood loss, and the ability to reveal the femoral head and neck. It can effectively scrape the anterior, anterolateral, and anteromedial necrotic bone tissue of the femoral head, facilitate the reconstruction of the lateral column of the femoral head and effectively support the subchondral bone. In addition, the subcutaneous soft tissue was isolated from the proximal end of the surgical incision, which could be cut directly from the iliac bone to be used as a bone grafting material, thereby reducing skin incision, decreasing pain, and improving patient satisfaction. When long-term joint preservation fails, the OCM approach can also be used for THA, avoiding additional surgical incisions, which may be more important for female patients. However, the limitation of head and neck bone grafting through the OCM approach is that it is difficult to access the necrotic area on the posterior and posterosuperior. Previous studies have reported the treatment of ONFH through the anterolateral approach, direct anterior approach, and surgical hip dislocation [20–22]. In our study, there was no comparison between OCM and other approaches in the treatment of femoral head necrosis. Further studies are needed to directly compare the efficacy.

Multiple studies have shown β-Tricalcium phosphate porous bioceramics degraded at a rate that matched the new bone formation [23–25]. Dai et al. [26] compared the use of β-tricalcium phosphate bioceramics and autologous iliac bone alone in lumbar fusion, and showed that β-tricalcium phosphate bioceramics as bone graft materials have similar performance to autologous bone. Zhang et al. [27] reported that β-tricalcium phosphate bioceramics and autologous iliac bone minimally invasive treatment of early and mid-term femoral head necrosis have shown satisfactory clinical effects. β-Tricalcium phosphate porous bioceramic bone is characterized by biocompatibility, good mechanical strength, degradation and absorption in vivo, and tissue conduction. In addition, it is non-toxic and non-carcinogenic, and its degradation product composition is close to the inorganic composition of human bone, which is conducive to the mineralization of bone matrix and has good osteoinductive properties. Appropriate amount of autologous bone filling to the necrotic area of the lesion can not only provide mechanical support to the bone grafting area, but also promote the formation of bone and blood vessels, effectively repairing bone defects. In this study, autologous iliac bone mixed with β-tricalcium phosphate porous bioceramic bone was selected as the bone graft.

Asser et al. [28] used inverted femoral head grafting via a lateral approach to the proximal femur for non-traumatic osteonecrosis of the femoral head, with 10-year postoperative survival rate of 67.3% and radiographic survival rate of 50.3%. However, for women and patients over 30 years of age, the risk of failure is higher, and the superolateral necrosis area is difficult to remove. Lin et al. [29] reported that the treatment of osteonecrosis of the femoral head using Lantern-shaped screw loaded with autologous bone was able to provide mechanical support to the femoral head with clinical success rate of 94% and radiographic success rate of 88%. But Lantern-shaped screw cannot be removed after bone remodeling, with inevitable disadvantages. Keizer et al. [30] used the Phemister technique in 78 hips with mean follow-up of 7 years. They mentioned clinical survival rate of 59% at an average 5-year postoperative period, which decreased to 44% at 10-year follow-up. Skeler et al. [13] reported that trapdoor technology used autologous bone combined with BMP-7, with an average follow-up time of 3 years, and the success rate was 67%. The trapdoor technology destroys the integrity of the articular cartilage on the femoral head, which may aggravate the damage of the femoral head and cause the cartilage to fail to heal after surgery. Fenestration of the head and neck was first reported by Rosenwasser [31], where the average follow-up time was 12 years and the success rate was about 81%. Yildiz et al. [20] performed head and neck fenestration via an anterolateral approach, and the clinical and radiographic success rates were 75 and 71%, respectively, at a mean follow-up time of 4.5 years, with a 100% Steinberg stage 4 failure rate. They believed that the femoral head appeared collapsed and surgical failure could not be avoided. Wang et al. [32] reported 68% success rate of head and neck fenestration combined with demineralized bone matrix and autogenous iliac bone graft in 138 cases of femoral head necrosis. In our study, head and neck fenestration via the OCM approach was performed to remove necrotic bone tissue. The cortical bone and cancellous bone of the autologous iliac bone are mixed with β-tricalcium phosphate porous bioceramic bone graft to improve the healing process of the femoral head, with a 63.83% hip preserving success rate and 36.17% functional excellent rate. About 78.72% of patients did not show radiographic progression postoperatively. The results of the current study suggest that clinical success is not entirely consistent with radiographic success.

Several studies have shown that bone repair response, especially excessive bone resorption in bone remodeling, may lead to secondary collapse. Vitamin D plays a major role in calcium absorption and bone health, which is involved in the regulation of bone and mineral metabolism and can effectively promote bone mineralization, increase intestinal absorption of calcium and phosphate, and has a direct effect on bone cells to optimize bone remodeling. Serum 25(OH)D in bone metabolism markers is an intermediate product of vitamin D metabolism and its level can reflect the nutritional status of vitamin D in the body [33–36]. CTX levels reflect osteoclastic bone resorption activity and are elevated to a similar extent as osteoclast hyperactivity and represent an important biochemical marker of bone resorption. Univariate analysis of data from this study identified 25(OH)D deficiency and preoperative ARCO stage as risk factors for postoperative clinical failure. The results of the Cox multivariate risk analysis suggested that stage IIIA was an independent risk factor for postoperative clinical failure. The postoperative clinical failure rate of 25(OH)D deficient patients was 51.52% and the postoperative clinical failure rates of patients with stages IIB, IIC, and IIIA were 10, 14.29, and 60.87%, respectively. Therefore, we believe that whether the patient’s femoral head collapses or not affects bone healing after surgery. For patients with 25(OH)D deficiency, increasing the level of vitamin D may be beneficial for bone healing after surgery and improve the clinical success rate.

This study has some limitations. First, prospective randomized controlled studies are lacking. All patients were admitted to the hospital after complete imaging tests to determine the surgical plan, which leads to inevitable bias. Second, the lack of a control group. No comparative study of different surgical approaches has been performed. Third, analysis of other bone metabolic markers is missing. Bone metabolic markers can be used to monitor dynamic changes between bone formation and bone resorption. Fourth, our follow-up period was relatively short and the sample size was relatively small, which requires increased follow-up time and expanded sample size.

## Conclusion

Fenestration of femoral head neck via OCM approach autologous bone mixed β-tricalcium phosphate porous bioceramic bone graft can effectively treat non-traumatic ONFH in the pre-collapse stage. However, for patients with 25(OH)D deficiency or ARCO in stage IIIA, this method has a high clinical failure rate. It may be necessary to seek other methods to improve the survival rate of the femoral head and delay the time to undergo THA.

## Data Availability

The datasets used or analyzed during the current study are available from the corresponding author on reasonable request.

## Abbreviations

OCM approach: Orthopdische Chirurgie München approach
ONFH: Osteonecrosis of the femoral head
THA: Total hip arthroplasty
ARCO: Association Research Circulation Osseous
BMI: Body mass index
25(OH)D: 25-Hydroxyvitamin D
CTX: Type I collagen carboxy-terminal peptide
